# A Clinical Study of Autologous Bone Marrow Mononuclear Cells for Cerebral Palsy Patients: A New Frontier

**DOI:** 10.1155/2015/905874

**Published:** 2015-02-18

**Authors:** Alok Sharma, Hemangi Sane, Nandini Gokulchandran, Pooja Kulkarni, Sushant Gandhi, Jyothi Sundaram, Amruta Paranjape, Akshata Shetty, Khushboo Bhagwanani, Hema Biju, Prerna Badhe

**Affiliations:** ^1^Department of Medical Services and Clinical Research, NeuroGen Brain & Spine Institute, Stem Asia Hospital and Research Centre, Sector 40, Plot No. 19, Palm Beach Road, Seawood (W), Navi Mumbai 400706, India; ^2^Department of Research & Development, NeuroGen Brain & Spine Institute, Stem Asia Hospital and Research Centre, Sector 40, Plot No. 19, Palm Beach Road, Seawood (W), Navi Mumbai 400706, India; ^3^Department of NeuroRehabilitation, NeuroGen Brain & Spine Institute, Stem Asia Hospital and Research Centre, Sector 40, Plot No. 19, Palm Beach Road, Seawood (W), Navi Mumbai 400706, India

## Abstract

Cerebral palsy is a nonprogressive heterogeneous group of neurological disorders with a growing rate of prevalence. Recently, cellular therapy is emerging as a potential novel treatment strategy for cerebral palsy. The various mechanisms by which cellular therapy works include neuroprotection, immunomodulation, neurorestoration, and neurogenesis. We conducted an open label, nonrandomized study on 40 cases of cerebral palsy with an aim of evaluating the benefit of cellular therapy in combination with rehabilitation. These cases were administered autologous bone marrow mononuclear cells intrathecally. The follow-up was carried out at 1 week, 3 months, and 6 months after the intervention. Adverse events of the treatment were also monitored in this duration. Overall, at six months, 95% of patients showed improvements. The study population was further divided into diplegic, quadriplegic, and miscellaneous group of cerebral palsy. On statistical analysis, a significant association was established between the symptomatic improvements and cell therapy in diplegic and quadriplegic cerebral palsy. PET-CT scan done in 6 patients showed metabolic improvements in areas of the brain correlating to clinical improvements. The results of this study demonstrate that cellular therapy may accelerate the development, reduce disability, and improve the quality of life of patients with cerebral palsy.

## 1. Introduction

Cerebral palsy (CP), a heterogeneous group of neurological disorders, is one of the most common physical disabilities observed in infants. It affects around 2 children per 1000. Several antenatal, perinatal, and postneonatal factors are responsible for CP which results in defect or lesion of the immature brain [1]. Majority of population of CP has spastic syndrome, movement restriction, cognitive impairment, and speech impairment amongst other complications [2].

CP is a permanent, nonprogressive neurological disorder that has no cure available. The standard approach aims at improving the child's functional abilities. It involves medications, physical therapy, occupational therapy, speech therapy, use of assistive device, and so forth. But these therapies in solitude are seldom effective [3] as they do not address the core pathology of neural tissue damage. Therefore, residual neurodeficits are common which affect the quality of life of the CP patients. Hence, an alternative therapeutic strategy needs to be evolved. Currently, cellular therapy has gained attention due to its neurorestoration abilities. Variety of cells comprising of embryonic stem cells, adult stem cells, umbilical cord blood cells, and induced pluripotent stem cells have been explored as a therapeutic option for neurological disorders including stroke, Alzheimer's and Parkinson's diseases, spinal cord injury, autism, and cerebral palsy [4–7]. Numerous preclinical studies have reported that these cells ameliorate the functional deficits in animal models of cerebral palsy [8, 9]. Amongst these, adult stem cells are preferred cell types as they do not involve any ethical or moral issues. The mechanism of action involves neuromodulation, neuroprotection, axon sprouting, neural circuit reconstruction, neurogenesis, neuroregeneration, neurorepair, and neuroreplacement [10, 11]. Delayed milestone is one of the major symptoms of cerebral palsy and cellular therapy may accelerate the developmental process in these children.

To demonstrate the therapeutic benefits of cell therapy in combination with rehabilitation, we carried out an open label, nonrandomized study on 40 cases which included all types of cerebral palsy. These children were administered autologous bone marrow mononuclear cells intrathecally. These cells were chosen as they are available abundantly, are easy to procure, and do not involve any complex processing. They are relatively safe and have no immunogenic complications compared to allogenic cells. This study demonstrates the safety, feasibility, and efficacy of the intervention. Effect of the intervention was evaluated on objective clinical and functional outcomes as they demonstrate the improvement in quality of life of patients with cerebral palsy.

## 2. Material and Methods

### 2.1. Ethics Statement

Patients were selected based on the World Medical Association Helsinki Declaration for Ethical Principles for medical research involving human subjects [12]. The protocol of the study was reviewed and approved by the Institutional Committee for Stem Cell Research and Therapy (IC-SCRT) in accordance with the Indian Council of Medical Research (ICMR) guidelines. This study was registered with ClinicalTrials.gov identifier NCT01978821. A written informed consent was obtained from the adult patients and parents of all the children. The intervention was explained to them in detail along with possible adverse events. The consent was also video recorded.

### 2.2. Study Design

Study was designed and conducted as an open label study in a single hospital centre starting from August 2010 to August 2013. The intervention included one dose of cell transplantation and standard neurorehabilitation. Cell therapy included intrathecal administration of autologous bone marrow mononuclear cells in 40 cases of cerebral palsy and neurorehabilitation included physiotherapy, occupational therapy, speech therapy, and psychological intervention. The procedure of cell transplantation was performed in one day for each case. This study evaluates the safety, feasibility, and efficacy of cellular therapy in combination with rehabilitation in children with cerebral palsy. The primary aim of the study was to evaluate the efficacy of intervention on these children for the period of 6 months. The secondary aim was to evaluate in detail the effect of intervention on different types of CP. Possible adverse events caused by the treatment were also monitored in this duration.

### 2.3. Patient Selection Criteria

40 cases of cerebral palsy, 26 males and 14 females, were included in the study. The age of the study group ranged from 17 months to 22 years. These cases consisted of three groups mainly diplegic CP, quadriplegic CP, and miscellaneous CP which included a mixture of dystonic CP, athetoid CP, and ataxic CP. The inclusion criteria were diagnosed cases of any type of CP and age above 12 months. The exclusion criteria were presence of acute infections, such as human immunodeficiency virus (HIV)/hepatitis B virus (HBV)/hepatitis C virus (HCV), malignancies, bleeding tendencies, pneumonia, renal failure, severe liver dysfunction, severe anemia (hemoglobin < 8), any bone marrow disorder, space occupying lesion in brain, and other acute medical conditions such as respiratory infection and pyrexia.

### 2.4. Intervention

#### 2.4.1. Preintervention Assessment

Before the intervention, all the patients underwent a detailed neuroevaluation along with serological, biochemical, and hematological tests. Magnetic resonance imaging (MRI) of the brain and electroencephalography (EEG) were also performed in all the patients. Gross Motor Function Classification System (GMFCS) was used to classify the severity of the disease. Positron emission tomography-computed tomography (PET-CT) scan was introduced at a later phase of the study.

#### 2.4.2. Procurement and Isolation of Autologous BMMNCs

Granulocyte colony stimulating factor (G-CSF) injections were administered 48 hours and 24 hours prior to the procedure. Bone marrow aspiration was carried out under general anesthesia. 80–100 mL of bone marrow depending on the age and body weight of the patient was aspirated from the anterior superior iliac crest using the bone marrow aspiration needle and was collected in heparinized tubes.

The mononuclear cells (MNCs) were separated from the aspirate using the density gradient method. The MNCs were checked for viability and count with propidium iodide dye in a TALI machine (Invitrogen). Average cell viability was found to be 98%. CD34+ counting was done by fluorescence activated cell sorting (FACS) using CD34 PE antibody (BD Biosciences).

#### 2.4.3. Transplantation of Bone Marrow Mononuclear Cells

The separated autologous bone marrow mononuclear cells (BMMNCs) were immediately injected intrathecally using an 18G Tuohy needle and epidural catheter between fourth and fifth lumbar vertebrae by medical experts. The average number of cells injected was 10.23 × 10^6^. Simultaneously, 20 mg/kg body weight methyl prednisolone in 500 mL Ringer lactate was given intravenously to enhance survival of the injected cells. Patients were then monitored for any procedure related adverse events.

#### 2.4.4. Neurorehabilitation

After the transplantation, all the patients were provided extensive neurorehabilitation for 4 days. A personalized home rehabilitation program was planned for each patient depending on the assessment done before the treatment. It included physiotherapy, occupational therapy, speech therapy, and psychological intervention. All patients were advised to continue the neurorehabilitation for at least 6 months.

#### 2.4.5. Outcome Measure

An extensive neuroevaluation of all the patients was carried out six months after the intervention. As a part of the protocol, video recording was performed before and after intervention to record functional improvements in the patients.

A grading system was devised to evaluate the functional outcome in every individual based on mild, moderate, and major improvements in the symptoms as follows:

 no improvement: improvement seen in less than 10% symptoms;
 mild improvement: improvement seen in 10–35% symptoms;
 moderate improvements: improvements seen in 35–70% symptoms;
 major improvements: improvements seen in more than 70% symptoms.

18-FDG PET-CT scan was introduced at a later phase of the study. It was performed before intervention and six months later to monitor the functional metabolic improvements in brain. PET was performed using the Siemens Biograph mct with 64-slice high speed scanner-3D PET True V wide detector (Siemens-CTI, Knoxville, Tennessee, USA), with an intrinsic resolution of 0.6 mm full width at half maximum (FWHM) and the images of 45–50 contiguous transverse planes with a field of view of 21.6 cm axial PET FOV with True V. Scenium Software was used to process the imaging data.

### 2.5. Statistical Analysis

McNemar's test was used to establish significance of association between the intervention and the improvement in symptoms. The degree of freedom was 1. The test was applied only to those symptoms which had number of affected patients ≥10.

Percentage analysis was done for all symptoms.

## 3. Results

Forty patients of cerebral palsy were included in this study. On a follow-up of 1 week, 3 months, and 6 months, all the patients underwent a detailed neurological evaluation. One week after the intervention, out of 40 CP patients, 6 had improvement in oromotor activities, 3 in neck control, 8 in sitting balance, 5 in standing and walking balance, and 6 in speech. Three months after intervention, 14 patients had improvement in oromotor activities, 11 in neck control, 17 in sitting balance, 15 in standing balance, 9 in walking balance, and 12 in speech. Overall, at six months, 38 out of 40 (95%) patients showed improvements and 2 did not show any improvement but they remained stable without any deterioration.

The total population was divided into types of CP for subgroup analysis. 11 were grouped as diplegic CP, 23 as quadriplegic CP, and 6 as miscellaneous type of CP (Table 1).

On the data of six months, a correlation between age, gender, and improvements (Table 2) was carried out along with a percentage analysis for every symptom in each group.

**Figure 1:**
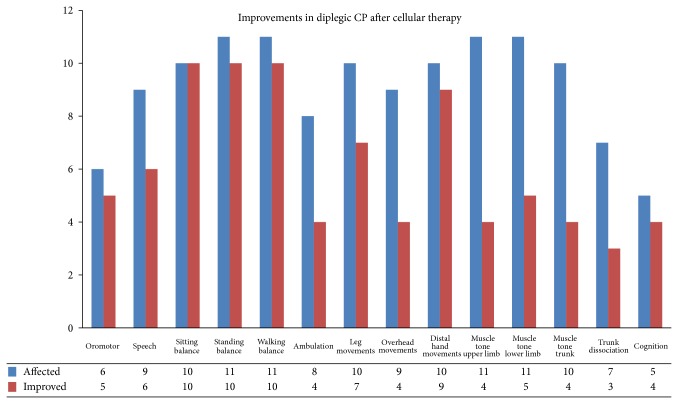
Improvements in diplegic CP: graph demonstrating symptomatic improvements in diplegic cerebral palsy after cellular therapy.

Amongst 11 diplegic CP patients, 100% showed improvement in sitting balance, 90.91% in standing and walking balance, 90% in distal hand movements, 83.33% in oromotor skills, 80% in cognition, 70% in leg movements, 66.67% in speech, 50% in ambulation, 45.45% in muscle tone of the lower limb, 44.44% in overhead activities, 40% in the muscle tone of the trunk, and 36.36% in muscle tone of the upper limb (Figure 1).

**Figure 2:**
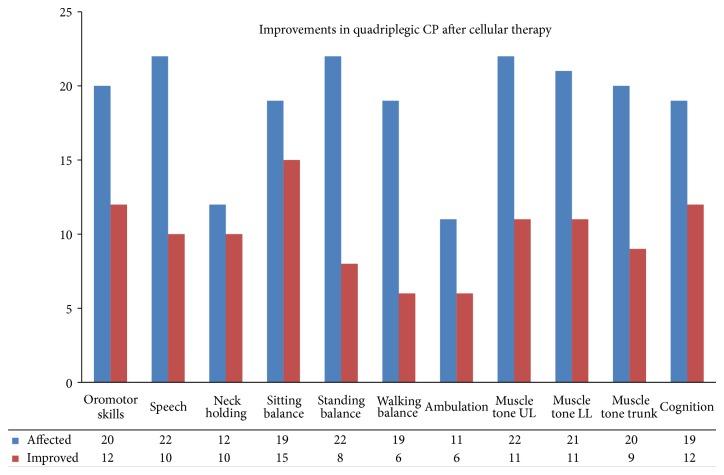
Improvements in quadriplegic CP: graph demonstrating symptomatic improvements in quadriplegic cerebral palsy after cellular therapy.

Amongst 23 quadriplegic CP, 83.33% showed improvement in neck holding, 78.95% in sitting balance, 63.16% in cognition, 60% in oromotor skills, 54.55% in ambulation, 52.38% in muscle tone of the lower limbs, 50% in muscle tone of upper limbs, 45.45% in speech, 45% in muscle tone of the trunk, 36.36% in standing balance, and 31.58% in walking balance (Figure 2).

**Figure 3:**
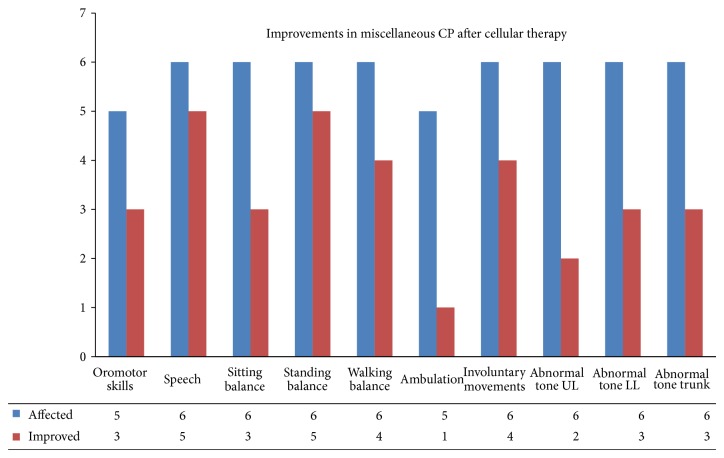
Improvements in miscellaneous group of CP: graph demonstrating symptomatic improvements in miscellaneous group of cerebral palsy after cellular therapy.

Amongst 6 cases in the miscellaneous group of CP, 3 were dystonic CP, 2 were athetoid CP, and 1 was ataxic CP. Overall, 83.33% showed improvement in speech and standing balance, 66.67% in walking balance, 60% in oromotor skills, 50% in sitting balance and muscle tone of lower limb and trunk, and 33.33% in muscle tone of upper limb. The patient with dystonic CP also showed an improvement in the dystonia (Figure 3).

Based on the grading system, we devised to evaluate the outcome of intervention; 7.5% cases showed no improvement, 17.5% cases showed mild improvement, 50% showed moderate improvement, and 25% showed major improvement.

**Figure 4:**
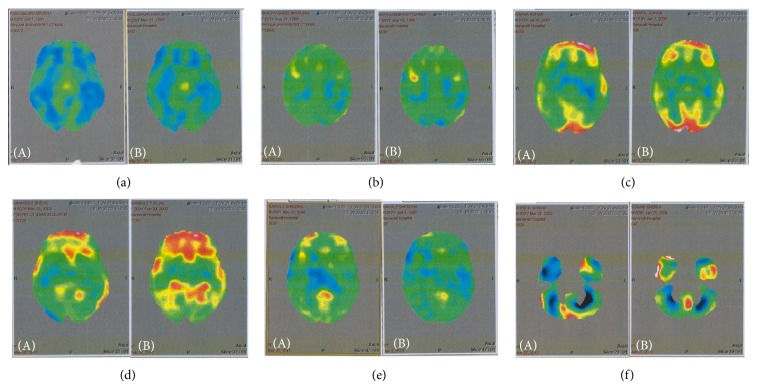
Improvements in PET-CT scan brain: PET-CT scan images of (A) pre- and (B) postintervention showing increased metabolic activity in various areas. Blue areas indicate hypometabolism, green areas indicate normal metabolism, yellow areas indicate slightly high metabolism, and red areas indicate high metabolism.

We further analyzed the improvements according to the baseline GMFCS level of all the patients (Table 3). Six patients chose to undergo the PET-CT scan to study the effect of intervention on the metabolic activity of the brain. On comparing the pre- and postscans, it was observed that the metabolism in areas such as frontal, temporal, parietal, basal ganglia, thalamus, and cerebellum had increased (Figure 4). The functional improvements observed in these patients also correlated with the areas of the brain showing change in metabolism.

### 3.1. Statistical Analysis

McNemar's test was performed on symptoms of diplegic and quadriplegic CP (where *N* ≥ 10) to find the significance of association between the improvement in the symptom and cell therapy. No statistical analysis was performed on miscellaneous group due to a very small sample size.

In diplegic CP, symptoms such as sitting, standing and walking balance, leg movements, and distal hand movements showed a statistically significant improvement after cell therapy (Table 4).

In quadriplegic CP, oromotor skills, speech, neck holding, sitting, standing and walking balance, ambulation, muscle tone of upper limb, lower limb, and trunk, and cognition showed a significant improvement after cell therapy (Table 5).

*Adverse Events.* At the time of the procedure, there were no complications recorded. But during the hospital stay, a few patients did show minor procedure related adverse events. 15% had a spinal headache, 7.5% had nausea, 30% experienced vomiting, 12.5% had pain at the site of injection, and 2.5% suffered diarrhea. These events were self-limiting and relieved within one week using medication. The only major adverse event noted related to cell transplantation was seizures which were observed in 2 patients (Table 6).

## 4. Discussion

Hypoxic ischemia is a common cause of damage to the fetal and neonatal brain leading to cerebral palsy and associated disabilities in children. The neuropathology underlying cerebral palsy mainly includes periventricular leukomalacia (PVL). PVL consists of diffuse injury of deep cerebral white matter, with or without focal necrosis [13] and/or loss of premyelinating oligodendrocytes (pre-OLs), astrogliosis, and microglial infiltration [14]. The vulnerability of these cells to damage depends on type of cells and stage of development at which the damage occurs [15]. Oligodendrocytes (OLs) develop through a well-established lineage of OL progenitors to pre-OLs to immature OLs to mature OLs. Loss of pre-OLs in hypoxic ischemia may lead to deficiency of mature OLs resulting in myelination disturbance which leads to neuronal dysfunctions [16, 17].

Another mechanism contributing to the pathophysiology of CP is the microglial activation after hypoxic ischemic injury. The microglia secretes various cytokines, such as tumor necrosis factor-alpha (TNF-*α*), interferon-gamma (INF-*γ*), interleukin-1 beta (IL-1*β*), and superoxide radicals, exerting a toxic effect on neurons and oligodendrocytes [18].

Owing to the heterogeneous nature of the pathophysiology of CP, standard medical interventions have a varied outcome. Recently, cell therapy is being developed as a treatment strategy for cerebral palsy [11, 19]. During childhood, the neuroplasticity of the brain is at maximum, rendering cellular therapy as a potent modality in children [19–23]. Various experimental studies have demonstrated that cell transplantation in the CP models lead to survival, homing, and differentiation of cells into neurons, oligodendrocytes, and astrocytes [24–26].

Stem cells stimulate the repair process by homing to the injured sites of the brain and carrying out regeneration [27]. Cell therapy restores the lost myelin by replacing the dead oligodendrocytes and their progenitors. It may also support their survival by introducing other cell types able to restore missing enzymes to an otherwise deficient environment [28]. Stem cells also reduce the levels of TNF-*α*, IL-1*β*, IL-1*α*, and IL-6 raised due to microglial activation, enhancing the endogenous brain repair [29]. These cells also secrete neurotrophic factors and growth factors such as connective tissue growth factor, fibroblast growth factors 2 and 7, interleukins, vascular endothelial growth factor (VEGF), fibroblast growth factor (FGF), and basic fibroblast growth factor (bFGF) which are responsible for cell proliferation, cytoprotection, and angiogenesis, retrieving the lost tissue functions [30, 31].

With an aim to study the safety, feasibility, and efficacy of cell therapy in cerebral palsy, we administered 40 cases with autologous bone marrow derived mononuclear cells. Studies have demonstrated that whole bone marrow mononuclear cells are a mixture of hematopoietic stem cells, mesenchymal stem cells, endothelial progenitor cells, macrophage, and lymphocytes. Together they exert a better effect compared to the individual fractions of the cells [32–34]. These cells were injected intrathecally as it is minimally invasive and safe and is an effective route of administration. Intracranial transplantation may be more targeted but it involves risk of surgical damage. In animal models of cerebral ischemia, it has been observed that, on intravenous administration, the majority of stem cells were found in organs other than brain such as lung, spleen, kidney, and intestine [35].

Along with cell therapy, all the patients also underwent rehabilitation as a part of the protocol. Majority of them were undergoing rehabilitation before the intervention but they still had major residual neurodeficits. Cell therapy and rehabilitation may together amplify the beneficial effects. Exercise promotes mobilization of stem cells, cell proliferation, and neurogenesis by increasing oxygen supply to the brain [36, 37].

All the 40 cases were followed up for duration of 6 months after intervention. After cell therapy, immediate improvements were observed within a week in muscle tone, involuntary movements of the limbs, head control, and drooling.

From 1 week to 3 months of intervention, improvement in voluntary control resulted in initiation of opening and closing of fingers and improved midline orientation. As the tone of the hypertonic muscles reduced, trunk control, sitting balance, and gross motor movements of limbs also improved. Normalization of muscle tone also helped to develop positive supporting reaction and integration of abnormal reflexes. Many patients also showed improved oromotor activities.

From 3 months to 6 months of intervention, eye hand coordination was better due to improved head control and gross motor skills. Sitting balance improved further along with initiation of weight shifting while sitting. Trunk dissociation movements were improved due to increase in trunk control and gross motor skills. Development of antigravity awareness helped in weight bearing on legs and maintaining an upright posture while standing. Initiation of steps while walking with support and/or assistive devices was also seen in nonambulatory patients. Cognitive skills improved progressively from one week to six months. Cooperation during therapy sessions was better, due to which it was easier for the caregiver to handle the patient. Muscle tone and motor control and independence for daily activities had improved. Cognition improved with respect to awareness, understanding, response time, and command following.

**Figure 5:**
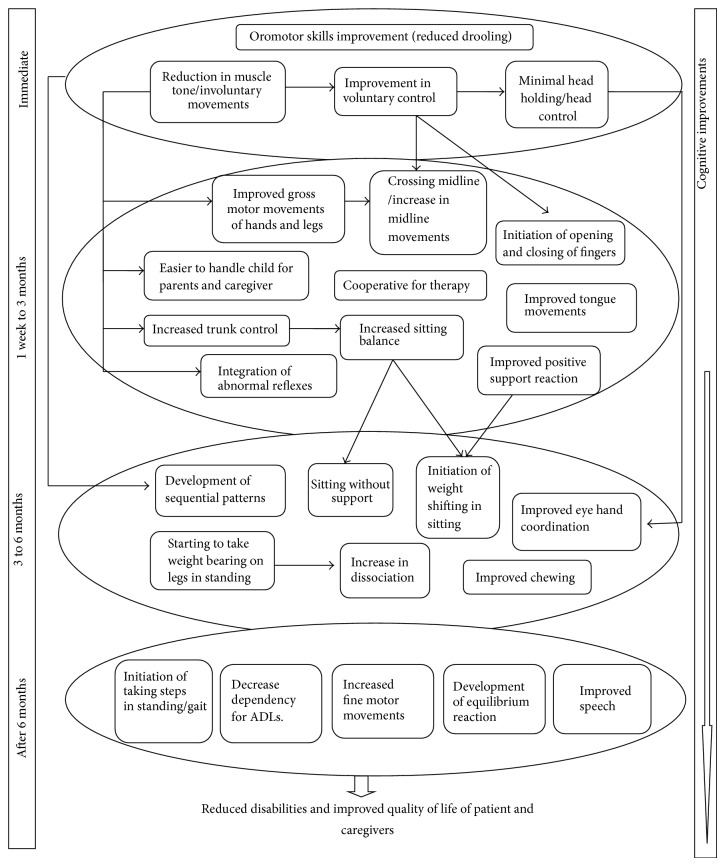
Flow chart depicting sequential developmental clinical improvements after autologous BMMNCs transplantation in cerebral palsy. On the left side, there are time periods within which the symptomatic improvements are seen. Each horizontal circle corresponds to the respective time period. The arrows signify direct causal effect between the symptomatic improvements. On the right side, the cognitive improvements are seen to be continuous.

Some patients were followed up even after six months. These patients showed improvement in fine motor activities. Equilibrium reactions developed along with increase in dynamic balance. Speech started improving in the aspects of clarity, fluency, and intelligibility. Individuals with monosyllable speech developed bisyllable speech, bisyllable improved to word formation, and words improved to phrases. There was also gradual improvement in ambulatory status (Figure 5).

18-FDG PET-CT scan was introduced at a later stage of the study to monitor improvements in the metabolic activity of the brain. The PET scan measures the 18-FDG uptake which correlates with the glucose metabolism in the brain. The damaged areas of the brain in CP are hypofunctioning and any improvement in the functioning of these areas will lead to an increase in the FDG uptake. Previous studies in patients with CP have shown reduced metabolic activity in various areas of the brain depending on the individual case [38]. A comparative scan performed before and after cell therapy demonstrated increased metabolic activity in frontal, parietal, temporal, basal ganglia, thalamus, and cerebellar areas of the brain. The clinical and functional improvements correlated with the changes observed in the PET scan. Improved metabolism in frontal and temporal areas led to improvement in speech and memory. Improvement in basal ganglia led to improved voluntary movement and coordination. Improvement in parietal area led to improved awareness and improvement in cerebellum led to improved balance and fine motor coordination (Table 7).

In CP, the development of milestones is delayed. As cellular therapy repairs the neural damage, it accelerates the development in these children. These improvements suggest that a combination of cell therapy and rehabilitation may lead to functional restoration which reduces disabilities in CP, thereby improving the quality of life of these patients.

*Limitations of This Study and Required Follow-Up Studies.* One of the major limitations of this study was that it was a nonrandomized open labeled study and did not have a placebo group to compare the outcome. There was also lack of a rehabilitation alone group which would have helped compare the effect of individual intervention. The duration of follow-up was short. A longer period of follow-up would be required to prove the efficacy of the intervention. Biomarker assays to correlate improvements were also lacking due to its cost and unavailability. The functional and behavioural assessments, after intervention, were not done blindly. The PET-CT scan as an outcome measure was introduced at a later stage so the effect was documented in only few cases. So, future studies may be planned with PET-CT scan as a monitoring tool.

This study indicates that autologous bone marrow mononuclear cell transplantation in combination with rehabilitation is safe, feasible, and efficacious. It may help to reduce the degree of impairment in cerebral palsy and improve the quality of life. Multicentric, large randomized controlled studies with long term follow-up, in future, will help to further substantiate the current outcome of the study. A lot of research about most potent cell types, routes of administration, number of dosages, target population, and time of intervention is still required to get the maximum benefits of cellular therapy. These future studies may help in advancement of cellular therapy as a treatment modality for cerebral palsy.

## Figures and Tables

**Table 1:** Demographical data (total number of patients = 40).

Demographic characteristics	Demographic group	Number of patients
Sex	Male	26
	Female	14

Age	<5 years	8
	5–10 years	16
	>10 years	16

Type of CP	Diplegic CP	11
	Quadriplegic CP	23
	Miscellaneous CP	6

GMFCS	Level I	2
	Level II	8
	Level III	6
	Level IV	9
	Level V	15

**Table 2:** Number of patients showing improvements based on gender and age of the patients 6 months after intervention.

	No improvement	Mild improvement	Moderate improvement	Significant improvement
Gender				
Male	3	4	14	5
Female	0	3	6	5
Age				
<5 years	0	1	5	3
5–10 years	1	3	8	3
>10 years	2	3	7	4

**Table 3:** Number of patients showing improvements based on the GMFCS levels of the patient six months after intervention.

GMFCS levels	Mild improvements	Moderate improvements	Major improvements
Level I	0	1	1
Level II	1	4	3
Level III	2	3	1
Level IV	1	6	2
Level V	6	6	3

**Table 4:** Statistical analysis for each symptomatic improvement in diplegic CP using McNemar's test.

Symptom	Number of patients affected	Number of patients improved	McNemar's test value	*P* value
Sitting balance	10	10	8.1	0.00443^*^
Standing balance	11	10	8.1	0.00443^*^
Walking balance	11	10	8.1	0.00443^*^
Leg movements	10	7	5.14286	0.02334^*^
Distal hand movements	10	9	6.125	0.00766^*^
Muscle tone upper limb (MAS)	11	4	2.25	0.13361
Muscle tone lower limb (MAS)	11	5	3.2	0.07364
Muscle tone trunk (MAS)	10	4	2.25	0.13361

^*^Significant at *P* value ≤ 0.05.

**Table 5:** Statistical analysis for each symptomatic improvement in quadriplegic CP using McNemar's test.

Symptom	Number of patients affected	Number of patients improved	McNemar's test value	*P* value
Oromotor	20	12	10.08333	0.0015^*^
Speech	22	10	8.1	0.00443^*^
Neck holding	12	10	8.1	0.00443^*^
Sitting balance	19	15	13.06667	0.0003^*^
Standing balance	22	8	6.125	0.01333^*^
Walking balance	19	6	4.16667	0.04123^*^
Ambulation	11	6	4.16667	0.04123^*^
Muscle tone upper limb (MAS)	22	11	9.09091	0.00257^*^
Muscle tone lower limb (MAS)	21	11	9.09091	0.00257^*^
Muscle tone trunk (MAS)	20	9	7.11111	0.00766^*^
Cognition	20	12	10.08333	0.0015^*^

^*^Significant at *P* value ≤ 0.05.

**Table 6:** Details of two patients who had seizures as an adverse event after cellular therapy.

Patient 1	The patient had abnormal EEG with generalised polyspike waves burst followed by background attenuation and multifocal epileptiform discharges and a history of seizures before intervention. After the intervention, the seizure frequency increased. The seizures stopped after 2 months by introducing Lamotrigine and increasing the dosage of Valproate.

Patient 2	The patient had abnormal EEG with sharp wave potentials over left parietal region and a history of seizures before intervention. After the intervention, the seizure frequency increased. This was controlled by increasing the dosage of Valproate and continuing Clobazam and levetiracetam.

**Table 7:** Describing the areas of the brain showing increased metabolism in the PET scan, carried out in six patients corresponding to the functional improvements.

Patient	Age/gender	Areas of the brain showing increased metabolism	Function improved
Patient 1	9/M	Left frontal, occipital, parietal, right basal ganglia, right temporal, thalamus	Speech, walking, balance, visual recognition, awareness, comprehension, cooperation, learning, memory, gross motor activities

Patient 2	16/F	Basal ganglia, cerebellum, frontal, parietal	Eye hand coordination, walking, balance, speech, memory, attention, concentration, learning

Patient 3	12/M	Right frontal and occipital, basal ganglia, parietal, temporal, thalamus	Fine motor activities, gross motor activities, movements, walking, balance, speech

Patient 4	23/F	Basal ganglia, bilateral frontal, parietal, temporal, thalamus	Speech, memory, hand movements, walking, balance

Patient 5	22/M	Right basal ganglia, cerebellum	Balance, walking, oromotor activities, comprehension, awareness, learning, grasping, memory, social interaction, coordination

Patient 6	3/F	Temporal, parietal, frontal, cerebellum, basal ganglia	Awareness, drooling, spasticity, balance

## References

[1] Longo M., Hankins G. D. V. (2009). Defining cerebral palsy: pathogenesis, pathophysiology and new intervention. *Minerva Ginecologica*.

[2] Odding E., Roebroeck M. E., Stam H. J. (2006). The epidemiology of cerebral palsy: incidence, impairments and risk factors. *Disability and Rehabilitation*.

[3] Sophie L. (2010). *Treatment of Cerebral Palsy and Motor Delay*.

[4] Sharma A., Sane H., Badhe P. (2012). Autologous bone marrow stem cell therapy shows functional improvement in hemorrhagic stroke: a case study. *Indian Journal of Clinical Practice*.

[5] Sharma A., Gokulchandran N., Sane H. (2013). Detailed analysis of the clinical effects of cell therapy for thoracolumbar spinal cord injury: an original study. *Journal of Neurorestoratology*.

[6] Sharma A., Gokulchandran N., Sane H. (2013). Autologous bone marrow mononuclear cell therapy for autism: an open label proof of concept study. *Stem Cells International*.

[7] Sharma A., Sane H., Paranjape A. (2013). Positron emission tomography-computer tomography scan used as a monitoring tool following cellular therapy in cerebral palsy and mental retardation—a case report. *Journal of Clinical Case Reports*.

[8] Rosenkranz K., Kumbruch S., Tenbusch M. (2012). Transplantation of human umbilical cord blood cells mediated beneficial effects on apoptosis, angiogenesis and neuronal survival after hypoxic-ischemic brain injury in rats. *Cell and Tissue Research*.

[9] Li Y., Tu L., Chen D., Jiang R., Wang Y., Wang S. (2012). Study on functional recovery of hypoxic-ischemic brain injury by Rg 1-induced NSCs. *Zhongguo Zhongyao Zazhi*.

[10] Woodbury D., Schwarz E. J., Prockop D. J., Black I. B. (2000). Adult rat and human bone marrow stromal cells differentiate into neurons. *Journal of Neuroscience Research*.

[11] Sharma A., Sane H., Paranjape A. (2013). Positron emission tomography—computer tomography scan used as a monitoring tool following cellular therapy in cerebral palsy and mental retardation—a case report. *Case Reports in Neurological Medicine*.

[12] Carlson R. V., Boyd K. M., Webb D. J. (2004). The revision of the Declaration of Helsinki: past, present and future. *British Journal of Clinical Pharmacology*.

[13] Folkerth R. D. (2005). Neuropathologic substrate of cerebral palsy. *Journal of Child Neurology*.

[14] Volpe J. J. (2003). Cerebral white matter injury of the premature infant—more common than you think. *Pediatrics*.

[15] Hagel C., Stavrou D., Panteliadis C. P., Strassburg H. M. (2004). Neuropathology of cerebral palsy. *Cerebral Palsy: Principles and Management*.

[16] Miron V. E., Kuhlmann T., Antel Jack J. P. (2011). Cells of the oligodendroglial lineage, myelination, and remyelination. *Biochimica et Biophysica Acta—Molecular Basis of Disease*.

[17] Susuki K. (2010). Myelin: a specialized membrane for cell communication. *Nature Education*.

[18] Hansson E., Rönnbäck L. (2003). Glial neuronal signaling in the central nervous system. *The FASEB Journal*.

[19] Sharma A., Gokulchandran N., Chopra G. (2012). Administration of autologous bone marrow derived mononuclear cells in children with incurable neurological disorders and injury is safe and improves their quality of life. *Cell Transplantation*.

[20] Mundkur N. (2005). Neuroplasticity in children. *The Indian Journal of Pediatrics*.

[21] Sharma A., Chopra G., Gokulchandran N., Lohia M., Kulkarni P. (2011). Autologous bone derived mononuclear transplantation in rett syndrome. *Asian Journal of Paediatric Practice*.

[22] Sharma A., Gokulchandran N., Badhe P. (2013). An improved case of autism as revealed by PET CT scan in patient transplanted with autologous bone marrow derived mononuclear cells. *Journal of Stem Cell Research & Therapy*.

[23] Sharma A., Gokulchandran N., Shetty A., Sane H., Kulkarni P., Badhe P. (2013). Autologous bone marrow mononuclear cells may be explored as a novel potential therapeutic option for autism. *Journal of Clinical Case Reports*.

[24] Qu S. Q., Luan Z., Yin G. C. (2005). Transplantation of human fetal neural stem cells into cerebral ventricle of the neonatal rat following hypoxic-ischemic injury: survival, migration and differentiation. *Zhonghua Er Ke Za Zhi*.

[25] Chen A., Siow B., Blamire A. M., Lako M., Clowry G. J. (2010). Transplantation of magnetically labeled mesenchymal stem cells in a model of perinatal brain injury. *Stem Cell Research*.

[26] Park K. I., Himes B. T., Stieg P. E., Tessler A., Fischer I., Snyder E. Y. (2006). Neural stem cells may be uniquely suited for combined gene therapy and cell replacement: evidence from engraftment of Neurotrophin-3-expressing stem cells in hypoxic-ischemic brain injury. *Experimental Neurology*.

[27] Alvarez P., Carrillo E., Vélez C. (2013). Regulatory systems in bone marrow for hematopoietic stem/progenitor cells mobilization and homing. *BioMed Research International*.

[28] Goldman S. A. (2011). Progenitor cell-based treatment of the pediatric myelin disorders. *Archives of Neurology*.

[29] Brenneman M., Sharma S., Harting M. (2010). Autologous bone marrow mononuclear cells enhance recovery after acute ischemic stroke in young and middle-aged rats. *Journal of Cerebral Blood Flow and Metabolism*.

[30] Gnecchi M., Zhang Z., Ni A., Dzau V. J. (2008). Paracrine mechanisms in adult stem cell signaling and therapy. *Circulation Research*.

[31] Daadi M. M., Davis A. S., Arac A. (2010). Human neural stem cell grafts modify microglial response and enhance axonal sprouting in neonatal hypoxic-ischemic brain injury. *Stroke*.

[32] Pösel C., Möller K., Fröhlich W., Schulz I., Boltze J., Wagner D.-C. (2012). Density gradient centrifugation compromises bone marrow mononuclear cell yield. *PLoS ONE*.

[33] Park H. C., Shim Y. S., Ha Y. (2005). Treatment of complete spinal cord injury patients by autologous bone marrow cell transplantation and administration of granulocyte-macrophage colony stimulating factor. *Tissue Engineering*.

[34] Brenes R. A., Bear M., Jadlowiec C. (2012). Cell-based interventions for therapeutic angiogenesis: review of potential cell sources. *Vascular*.

[35] Steiner B., Roch M., Holtkamp N., Kurtz A. (2012). Systemically administered human bone marrow-derived mesenchymal stem home into peripheral organs but do not induce neuroprotective effects in the MCAo-mouse model for cerebral ischemia. *Neuroscience Letters*.

[36] van Praag H., Christie B. R., Sejnowski T. J., Gage F. H. (1999). Running enhances neurogenesis, learning, and long-term potentiation in mice. *Proceedings of the National Academy of Sciences of the United States of America*.

[37] Colcombe S., Kramer A. F. (2003). Fitness effects on the cognitive function of older adults: a meta-analytic study. *Psychological Science*.

[38] Kannan S., Chugani H. T. (2010). Applications of positron emission tomography in the newborn nursery. *Seminars in Perinatology*.

